# *Aronia melanocarpa* Treatment and Antioxidant Status in Selected Tissues in Wistar Rats

**DOI:** 10.1155/2014/457085

**Published:** 2014-06-05

**Authors:** Renata Francik, Mirosław Krośniak, Ilona Sanocka, Henryk Bartoń, Tomasz Hebda, Sławomir Francik

**Affiliations:** ^1^Department of Bioorganic Chemistry, Medical College, Pharmacy Faculty, Jagiellonian University, 9 Medyczna Street, 30-688 Krakow, Poland; ^2^Institute of Health, State Higher Vocational School, Staszica 1 Street, 33-300 Nowy Sącz, Poland; ^3^Department of Food Chemistry and Nutrition, Medical College, Pharmacy Faculty, Jagiellonian University, 9 Medyczna Street, 30-688 Krakow, Poland; ^4^Department of Mechanical Engineering and Agrophysics, Faculty of Production Engineering and Energetics, University of Agriculture in Krakow, 116 B Balicka Street, 30-149 Krakow, Poland

## Abstract

*Aronia* juice is considered to be a source of compounds with high antioxidative potential. We conducted a study on the impact of compounds in the *Aronia* juice on oxidative stress in plasma and brain tissues. The influence of *Aronia* juice on oxidative stress parameters was tested with the use of a model with a high content of fructose and nonsaturated fats. Therefore, the activity of enzymatic (catalase, CAT, and paraoxonase, PON) and nonenzymatic (thiol groups, SH, and protein carbonyl groups, PCG) oxidative stress markers, which indicate changes in the carbohydrate and protein profiles, was marked in brain tissue homogenates. Adding *Aronia* caused statistically significant increase in the CAT activity in plasma in all tested diets, while the PON activity showed a statistically significant increase only in case of high fat diet. In animals fed with *Aronia* juice supplemented with carbohydrates or fat, statistically significant increase in the PON activity and the decrease in the CAT activity in brain tissue were observed. In case of the high fat diet, an increase in the number of SH groups and a decrease in the number of PCG groups in brain tissue were observed.

## 1. Introduction

High demand and consumption of oxygen, along with increased mitochondrial density, result in the particular sensitivity of nerve tissue to oxidative stress. In mitochondria, as a consequence of oxidative metabolism, oxygen is reduced to water. The by-products of this reaction include superoxide radical and hydroxyl radical, which are produced in excessive amounts and can lead to the disruption of homeostasis in nervous tissue [1]. High concentration of polyunsaturated fatty acids (PUFA) in membranes of nerve cells can be the reason for the formation of excessive amounts of reactive oxygen species (ROS) in nervous tissue. In the presence of free radicals, lipids undergo peroxidation. Moreover, ROS can also be formed as a result of excessive activation of neutrophils and elevated metabolism of arachidonic acid or catecholamines. Additionally, low level of albumin in the cerebrospinal fluid can increase the pool of free radicals [1]. What protects the brain tissue of an organism from destructive effects of ROS is the system of enzymatic and nonenzymatic antioxidants called antioxidant barrier. Superoxide dismutase, catalase, glutathione peroxidase, glutathione reductase, and glutathione S-transferase compose the group of enzymatic factors. The group of nonenzymatic factors consists of ceruloplasmin, transferrin, melatonin, albumin, polyamides, the transition metals (zinc, copper, and selenium), glutathione, thioredoxin, as well as vitamins (A, C, and E), and polyphenolic compounds [2].

Vegetables and fruits are a source of both nutrients and substances devoid of nutritional value, most of which act as antioxidants. At the beginning of the 1990s, red wine was a subject of intense studies. Health oriented properties of this drink were called “the French paradox.” On the basis of available studies, anthocyanins were proven to be responsible for therapeutic properties of red wine. Anthocyanins demonstrate strong antioxidant properties. Compared to vitamin C, recognized as the reference substance, these values are approximately 700 times higher [3]. These compounds create tissue protection against the detrimental effects of free radicals. Observations concerning the properties of flavonoid derivatives, pertaining to the group of nonenzymatic antioxidants, are significant in the pathophysiology of many diseases, including diabetes, Alzheimer's disease, heart disease, and rheumatoid arthritis [4]. Currently, studies on plants rich in these compounds, such as* Aronia*, are being conducted [5]. These materials demonstrate high concentration of polyphenolic compounds. Anthocyanins from* Aronia melanocarpa* may be of benefit to patients with MS as far as atherosclerosis prevention is concerned. It seems to result from anthocyanins' influence on blood pressure, ET-1 level, serum lipids, and oxidative status [6].

*Aronia*, which belongs to the rose family (Rosaceae), is one of the plants exhibiting considerable antioxidant potential. This is the result of the content of polyphenolic compounds, such as anthocyanins (cyanidin 3-O-galactoside, cyanidin 3-O-arabinoside, cyanidin 3-O-xyloside, and cyanidin 3-O-glucoside), flavonoids (quercetin 3-O-vicianoside, quercetin 3-O-robinobioside, and other quercetin glucosides), and phenolic acids (chlorogenic acid, neochlorogenic acid, caffeic acid, and ferulic acid) along with vitamins C and E [7].* Aronia* has been used as a dietary supplement in cases of cancer and as anti-inflammatory or antiulcer drug [8]. A positive impact of* Aronia* on animals with experimentally produced diabetes has also been tested. During the study, normalisation of body weight and of biochemical parameters of diabetes (including a decrease in glucose level in blood and urine) along with reduction of thirst and amount of urine passed has been observed [9].

Epidemiological studies have shown that dietary habits can influence the incidence of Alzheimer's and Parkinson's diseases [10–12]. Foods, and especially components chemically classified as antioxidants (in green tea polyphenols in particular), have been reported to have a beneficial effect in neuroprotection [13, 14].

There are many scientific premises concerning influence of free radicals and substances causing oxidative stress on neuroprotective diseases. One of the ways to induce oxidative stress in animals is to provide food supplemented with fat and high amounts of fructose. To assess the antioxidative status of brain tissue, a model based on fructose and high fat diets was used. Diet is a major factor in maintaining neural and cognitive health throughout the lifespan, and changes in diet and lifestyle have promoted an epidemic of obesity and related health problems all over the world. Nowadays, poorly composed diet is a factor causing many lifestyle diseases in people, especially in the elderly. For example, the diet rich in monosaccharides or saturated fats is the cause of accelerated dementia development in the elderly people in Alzheimer's disease [15, 16].

Due to the low glycemic index, fructose was recommended to patients with diabetes. The research has shown that food with a high content of fructose may increase the triglyceride level in plasma as well as cause hyperinsulinaemia, insulin resistance, increased blood pressure, and heart diseases [17, 18]. In the conducted research, the influence of the dosage of* Aronia* juice on the changes caused in brain tissue by the increased amount of fructose and saturated fats was assessed [19].

Compounds present in* Aronia* juice are a rich source of anthocyanins and polyphenolic substances, all reducing the amount of free radicals. The aim of our study was to determine the oxidative status of plasma and brain tissue in Wistar rats. The animals were fed control (C−), fructose (F−), and high fat (Fa−) diet and provided with drinking water without the addition of* Aronia* juice or water with this juice added (groups C+, F+, and Fa+). Brain tissue was selected for marking due to its particular vulnerability to oxidative stress which may be the cause of many diseases. In patients suffering from Alzheimer's disease or stroke, it is essential to introduce a proper diet which, through appropriate components, will lead to health improvement [20]. Bodily lipids, proteins, glycoproteins, and nucleic acids are subject to oxidative injury, and a number of analytical methods exist for measurement of oxidative by-products in blood and brain tissue samples. The measure of oxidative stress was expressed by paraoxonase (PON1) and catalase (CAT) activity, and (FRAP). The total capacity of antioxidant of tissue was of expressed by protein carbonyl group (PCG), sulfhydryl group (SH).

## 2. Materials and Methods

### 2.1. Animals

In the experiment, male Wistar rats aged 3 months and weighing 250 ± 15 g were divided into 6 groups of 6 animals. For 5 weeks, the animals were given control feed (group C), fructose feed (group F), and high fatty feed (Fa) (Table 1). For these groups of animals, which was not provided* aronia* juice we introduced markings C−, F− and Fa−.

The animals from groups C+, F+, and Fa+ were administered* Aronia* juice mixed with water in a volume ratio of 3 : 1 (v/v). The juice was produced by the Eko-Ar company (it was 100% cold-pressed* Aronia* juice). Moreover, all animals had free access to feed and water. They were kept in a room with a constant temperature of 23°C and 50–60% humidity with a 12-hour day/night cycle. After 5 weeks, they were euthanized by intraperitoneal injection of sodium thiopental 60 mg/kg. Blood samples were taken from the aorta into heparinized tubes and then centrifuged (at 3000 ×g for 15 minutes at 4°C) to obtain plasma that was immediately analyzed or kept frozen at −80°C until the time of further analyses. Brain tissue was rapidly removed, weighed, and immediately frozen in liquid nitrogen and stored at −80°C until further analyses. The experiments were performed in compliance with the requirements of the Local Commission of Ethics in Krakow.

### 2.2. Tissue Preparation

Brain tissue was minced in 0.15 M phosphate buffer, pH = 7.4 to 5% final concentration using a basic ultraspeed tissue grinder, the Ultra Turrax T25 homogenizer (12000 r/min bursts). All procedures were performed on ice. Homogenized tissues were centrifuged at 1000 ×g for 15 min (0–4°C). The resulting supernatant was drawled and the pellet was discarded.

### 2.3. Measurement of Protein Carbonyl Group (PCG)

Protein carbonyl group (PCG) content was measured by the method of Levine et al. [21]. Protein was precipitated with 20% trichloroacetic acid (TCA). After centrifuging at 11000 ×g and at 4°C for 15 min, the supernatant was removed. The pellet was resuspended in 0.5 mL of 10 mM 2,4-dinitrophenylhydrazine (DNPH)/2 M HCl. Samples were held in a dark place for 1 h and then vortexed for 10 min. The samples were precipitated with 0.5 mL of 20% TCA and centrifuged at 11000 ×g and at 4°C for 3 min. The same procedure was repeated with 10% TCA for three times. Precipitate was dissolved in 2 mL of 8 M urea at 37°C. The carbonyl group content was determined by measuring the absorbance at 370 nm. Results are expressed in nmoles of carbonyl per mg of soluble protein.

### 2.4. Measurement of Ferric Reducing Antioxidant Power (FRAP)

The FRAP method has been used in antioxidant properties measurements. In acidic environment, Fe^3+^ present in FRAP is reduced to Fe^2+^, possessing intensive blue color, with maximum absorbance at 593 nm. The FRAP is the modification of Benzie and Strain's method [22]. In case of the FRAP method, the Fe^2+^ content in the tested samples of homogenate brain and plasma was calculated based on the standard curve. The FRAP concentration values (mM) for the tested substances were read in the 15th minute of the test.

### 2.5. Measurement of Sulphydryl Group (SH)

Total sulphydryl contents were determined using 5,5′-dithiobis-(2-nitrobenzoic acid) (DTNB) according to Ellman's method [23] with some modifications: 50 *μ*L of the homogenate brain or plasma was mixed with 1 mL of 0.05 M phosphate buffer pH 7.2 containing 0.6 M NaCl, 6 mM ethylenediaminetetraacetic acid (EDTA), and 8 M urea. The mixture was centrifuged for 15 min at 14000 ×g at 5°C. To 3 mL of the supernatant, 0.04 mL of 0.01 M DTNB solution in 0.05 M sodium acetate was added and incubated at 40°C for 15 min. A blank was prepared replacing the homogenate with 0.05 M phosphate buffer pH 7.2 containing 0.6 M NaCl, 6 mM EDTA, and 8 M urea. The absorbance was measured at 412 nm and the SH content was calculated using a molar extinction coefficient of 13600 M^−1^ cm^−1^. Results were expressed in micromoles of SH per mg of protein.

### 2.6. Measurement of Paraoxonase Activity (PON1)

Paraoxonase enzyme activity was determined using our own modification of Eckerson et al. [24] method. Paraoxonase activities measurements were performed in the presence of NaCl (salt-stimulated activity). The rate of paraoxon hydrolysis (diethyl-p-nitrophenyl phosphate) was measured by monitoring an increase in absorbance at 412 nm at 25°C. The amount of generated p-nitrophenol was calculated from the molar absorptivity coefficient at pH 8.0 which was 18290 M^−1^ cm^−1^. Paraoxonase activity was expressed as U/mg of protein.

### 2.7. Measurement of Catalase (CAT) Activity

The activity of catalase (CAT) was estimated in the brain tissue homogenates and plasma. The catalase activity was measured by Aebi's method [25]. The measurements were performed spectrophotometrically at 240 nm at 25°C. One unit of CAT activity was defined as the amount of enzyme decomposing 1 *μ*mol of H_2_O_2_ per minute. CAT concentrations were expressed in U/mg of protein.

### 2.8. Statistical Analysis

The results in this study were presented as mean values ± standard deviations (SD). Normality of all of the dependent variables (PCG, FRAP, SH, and PON CAT) was tested using the Shapiro-Wilk test. Statistical differences between the diets with and without* Aronia* supplement were analyzed by the two-way ANOVA test with PCG, FRAP, SH, and PON CAT difference as the dependent variables and Diet,* Aronia*, and Diet∗*Aronia* as effects. Differences were regarded as significant at risk levels of *P* < 0.05. Tukey's HSD (honestly significant difference) test was applied to assess significant differences (*P* < 0.05) between samples. Statistical analyses were performed with STATISTICA PL software, version 10 (StatSoft, Inc.).

### 2.9. Reagents

All chemicals, solvents, and standards of reagents used in experiments were produced by Sigma-Aldrich. Double-distilled deionized water (Milli-Q, Millipore 18.2 MW/cm 25°C) was used in all experiments.

## 3. Results and Discussion

When oxidants exceed the antioxidant defense, biological systems suffer oxidative stress, with damage to biomolecules and functional impairment. The brain is inherently sensitive to oxidative stress due to higher energy requirement, higher amounts of lipids, iron, and autooxidizable catecholamines, and lower levels of certain endogenous antioxidant molecules [26, 27]. In comparison to other berries,* Aronia* contains high amounts of biologically active hydroxycinnamic acids, chlorogenic acid (35.5 mg/100 g) and neochlorogenic acid (21.5 mg/100 g) [28]. Its influence inhibiting the *γ*-aminobutyric acid (GABA) transformation in the central nervous system as well as the immunostimulating properties has been proven. Additionally,* Aronia* shows the ability to reduce the concentration of lipids and oxygen free radicals and inhibits the release of histamine [29].

Carbonyl groups (PCG) are a relatively new marker of oxidative stress and they are used to assess changes in proteins. They can be applied as a biomarker in patients with diabetes. In this disorder, a rise in the concentration of PCG has been observed [30]. The summary of the results is presented in Table 2 (mean values ± standard deviation).

The variance analysis conducted for the PCG values obtained in brain tissue did not show a statistically significant influence of any of the independent variables (Diet,* Aronia*);* Aronia* juice did not have impact on the value of this parameter in the brain tissue homogenate in the presence of carbohydrates or increased amount of lipids, which may be caused by a positive influence of flavonoids in this juice.

**Figure 1:**
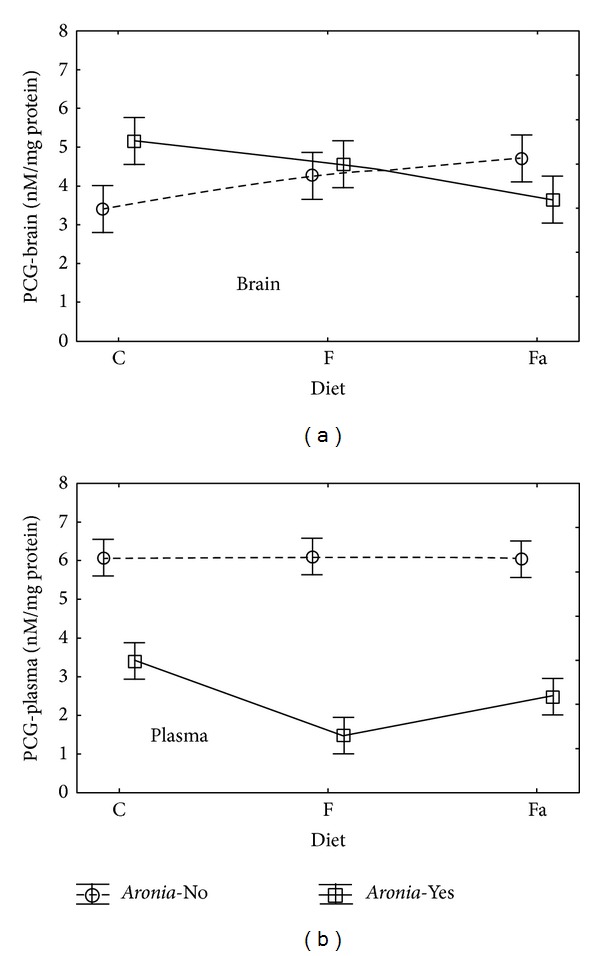
Interaction of factors diet and* Aronia* mean (±SEM) PCG concentration in brain and plasma for Wistar rats.

In plasma, however, adding* Aronia* juice to water showed a significant influence on the PCG value. In the animals fed the fructose diet with* Aronia* (F+), the highest decrease in the PCG amount was observed (Table 2, Figure 1). In case of the Fa+ and C+ groups, supplementing the diet with* Aronia* also caused a statistically significant decrease in the PCG amount.

The concentration of PCG reflects not only oxidative modifications of proteins but indirectly also lipid and carbohydrate changes induced by oxidative stress [31]. The creation of protein carbonyl derivatives is a comparatively complicated and lengthy process and, therefore, their presence constitutes an indicator of serious oxidative damage of the body [32]. Based on the research results, one can conclude that* Aronia* has protective function with regard to plasma.

**Figure 2:**
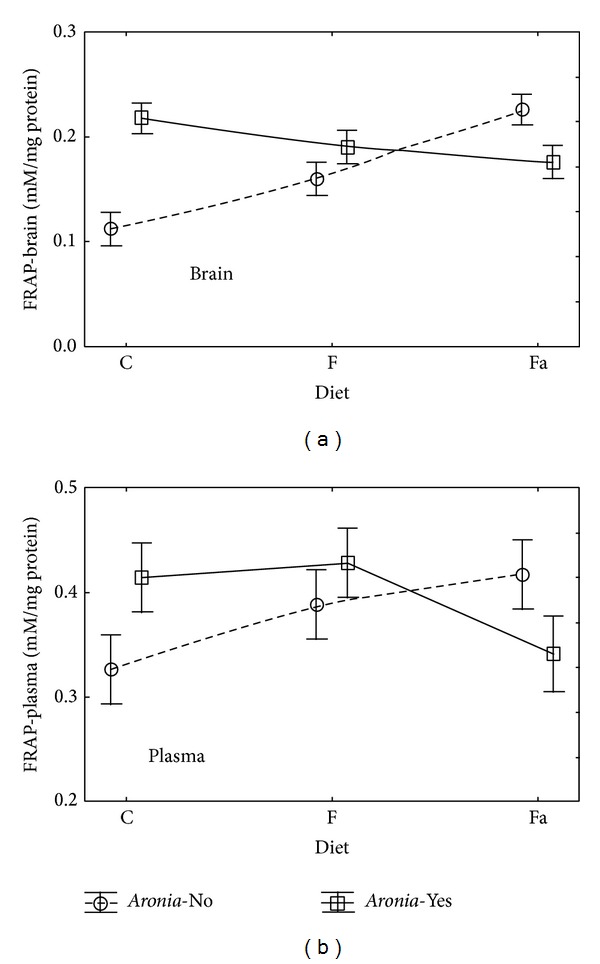
Interaction of factors diet and* Aronia* mean (±SEM) FRAP level in brain and plasma for Wistar rats.

In case of the evaluation of total antioxidant capacity expressed as FRAP for brain tissue homogenate (Table 2 and Figure 2), it was noted that adding* Aronia* to water had statistically significant influence on FRAP (*P* = 0.0307). One cannot, however, reject the hypothesis that the type of diet has no influence on the FRAP-brain value (*P* = 0.0696). Joint action of the tested factors with high statistical significance (*P* = 0.0001) was observed. In the animal group with the control diet (C−), the FRAP-brain value was on average 0.11 mM Fe^2+^/mg protein whereas adding* Aronia* (C+) doubled the FRAP-brain value 0.22 mM Fe^2+^/mg protein (Figure 2). In the animal group fed the fructose diet, the FRAP-brain value increase due to* Aronia* addition was lower (from 0.16 to 0.19 mM Fe^2+^/mg protein). Adding* Aronia* to water caused the decrease in FRAP-brain value only in the animal group fed high fat diet.

**Figure 3:**
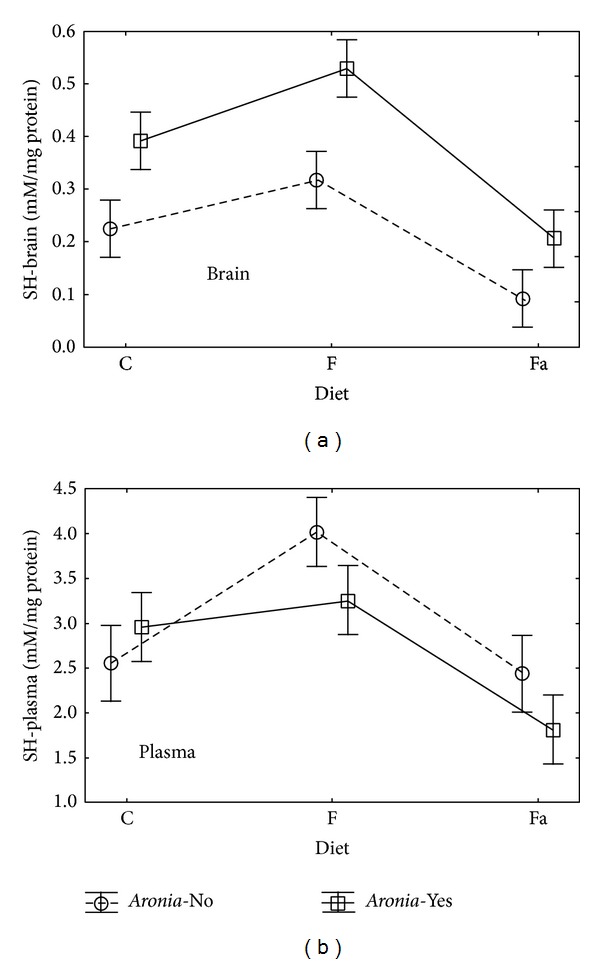
Interaction of factors diet and* Aronia* mean (±SEM) SH level in brain and plasma for Wistar rats.

In case of plasma, no statistically significant influence of the diet type or the* Aronia* addition on the FRAP value was observed. Mean values of that parameter in plasma were higher than in the brain homogenate and fluctuated in the range from 0.33 to 0.43 mM Fe^2+^/mg protein. Within that range, adding* Aronia* in the C+, Fa+ and F+ groups caused an increase of the FRAP value in plasma, similarly as in the brain homogenate (Figure 2). As regards the number of sulfhydryl groups in brain tissue (SH-Brain, Figure 3), both the diet type (*P* = 0.0001) and the* Aronia* addition (*P* = 0.0008) showed statistically significant influence (ANOVA *P* < 0.05). At the same time, no statistically significant interaction of the tested parameters (Diet∗*Aronia*) was observed. Adding* Aronia* to water doubled the SH-brain value (Figure 3) in case of the control diet (C−) from 0.23 to 0.39 mM/mg protein, in case of the fructose diet (F−) from 0.32 to 0.53 mM/mg protein, and in case of the high fat diet from 0.09 to 0.21 mM/mg protein.

In case of plasma, only the diet type has statistically significant influence on the SH value (*P* = 0.0027). SH-plasma mean values for individual groups were ten times higher than the SH-brain and they ranged from 1.81 to 4.02 mM/mg protein. The lowest SH-plasma values were observed for the high fat diet with* Aronia* (Fa+) and they were 1.81 nM/mg protein. In case of this diet,* Aronia* caused a reduction of the number of SH groups (2.44 nM/mg protein for the Fa group without* Aronia*). The highest SH-plasma values occurred in the fructose diet with* Aronia* (4.02 nM/mg protein) and the fructose diet without* Aronia* (3.26 nM/mg protein). As in the previous case, the decrease in the number of SH groups in animals fed with* Aronia* juice was observed.

Sulphydryl groups (SH) are components of compounds with antioxidative properties (glutathione peroxidase, albumin). Undergoing oxidation to disulfide bridge, they reflect the loss of compensatory capacity of antioxidant mechanisms [33]. SH groups protect cells against damage caused by free radicals, as they participate in maintaining an adequate structure and function of proteins, in regulating the enzymatic activity [34, 35]. The research conducted by Kaviarasan et al. [36] shows that the polyphenol extract from fenugreek increases the SH group level in the rat liver. Hininger-Favier et al. [37] did not observe a significant influence of polyphenols in green tea on the SH group concentration in the liver of rats fed with high carbohydrate diet. Based on the conducted research, a statistically significant influence of* Aronia* on the SH group number in the brain tissue can be observed. This observation does not, however, apply to plasma.

A fall in the concentration of SH groups in diabetes is an effect of oxidative stress, which is associated not only with elevated oxidation of proteins but also with increased glycation. Administered supplements have, therefore, reduced the degree of protein oxidation [30].

Oxidative modifications of proteins are the fastest emerging indicator of cells oxidative damage, demonstrating redox balance disturbance. This is due to the fact that they are not only substrates for chemical reactions but also catalysts for multiple processes in the body [38]. Therefore, changes in their structure and modifications in their function are much more important than with other biomolecules.

The main role in the development of atherosclerosis is attributed to oxidative modification of LDL inside the vascular wall. This process stimulates the release of numerous proinflammatory substances that can initiate the process of atherosclerosis. Experimental studies have shown that HDL inhibits the oxidative modification of LDL by detoxification of oxidized phospholipids produced during lipid peroxidation. This antioxidant effect is possible due to antioxidant properties of enzymes such as paraoxonase 1 (PON1) and/or platelet-activating factor acetylhydrolase (PAF-AH) [39].

PON1 is one of the key enzymes involved in antioxidant defense mechanism. Due to commonly undertaken research on the importance of PON1 activity in disease processes and the existence of a relationship between changes in enzyme activity and gene polymorphism, a possibility to develop effective diagnostic tests will arise in the nearest future [40, 41]. The variance analysis conducted for the paraoxonase activity (PON1) showed a significant influence of diet with* Aronia* addition on the brain tissue (PON1-brain; *P* = 0.00006), while for plasma (PON1-plasma) only the interaction of the tested variables was statistically significant (Diet∗*Aronia*; *P* = 0.0029).

**Figure 4:**
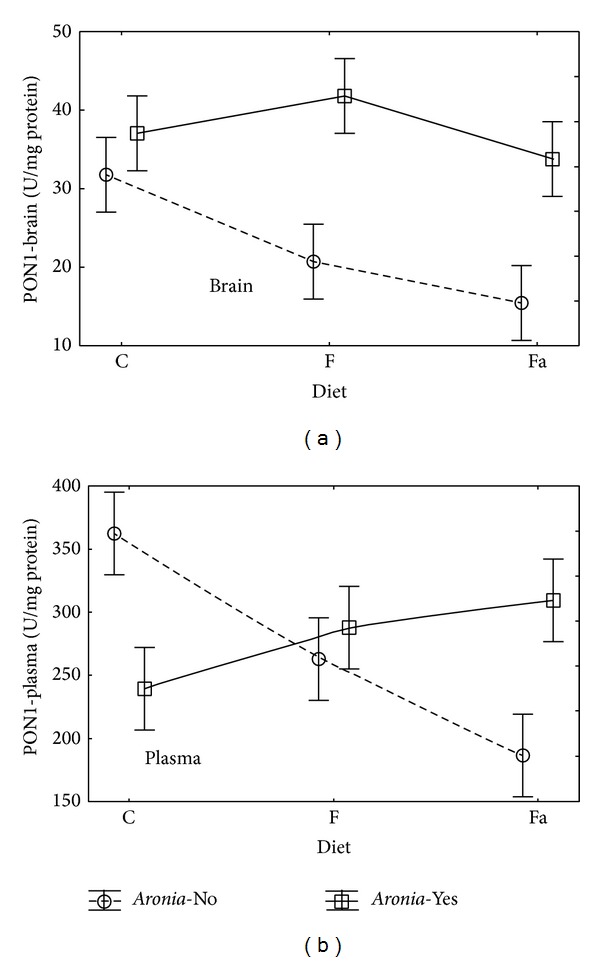
Interaction of factors diet and* Aronia* mean (±SEM) PON1 activity in brain and plasma for Wistar rats.

In brain, adding* Aronia* to water increased the PON1 activity value (Figure 4). In the animal group fed with the control diet with* Aronia*, the mean value increased in comparison to the C group to 37.1 (from 31.8) U/mg protein. In case of the fructose diet (F−) and high fat diet (Fa+), a statistically significant increase in the PON1-brain mean value occurred from 20.7 to 41.9 U/mg protein and from 15.5 to 33.8 U/mg protein, respectively. Paraoxonase activity marked for plasma (PON1-plasma) assumes nearly ten times higher values than the one marked for brain. In case of the control diet, the addition of* Aronia* decreased the PON1-plasma mean value from 363 to 240 U/mg protein, while in case of the fat diet the PON1 mean value increased from 187 to 309 U/mg protein. In case of the fructose diet, the differences between the group with the* Aronia* addition in water (*Aronia*-Yes) and the group without* Aronia* addition (*Aronia*-No) were not significant.

Being a bifunctional hemoprotein, catalase can act as catalase and peroxidase. In the environment of high hydrogen peroxide concentration, catalytic action prevails, involving the catalysis of reaction of hydrogen peroxide dismutation to molecular oxygen and water [42]. Catalase, as an enzyme involved in the catabolism of hydrogen peroxide and exogenous substrates, plays a role in the pathophysiology of diseases with associate inflammation. Numerous diseases, including pneumonia, tuberculosis, atherosclerosis, diabetes, hepatitis, cancer, neurodegenerative diseases (Parkinson's disease, Alzheimer's disease), and nephritis, are accompanied by the reduction of catalase activity [43–47].

In case of brain tissue, the catalase activity (CAT-brain) is statistically significantly influenced by both main factors, that is, the diet type (Diet: *P* = 0.0001) and adding* Aronia* to water (*Aronia*: *P* = 0.0001). There is no statistically significant interaction between these variables. The catalase activity for plasma (CAT-plasma) depends on the* Aronia* addition (*Aronia*: *P* < 0.0001). The interaction of the tested variables is also significant (Diet∗*Aronia*; *P* < 0.0001).

**Figure 5:**
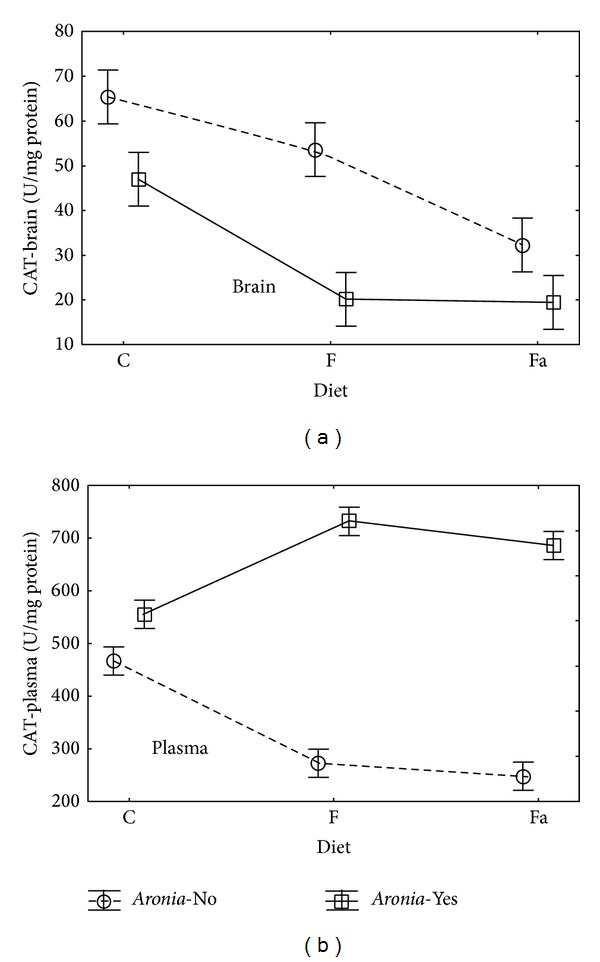
Interaction of factors diet and* Aronia* mean (±SEM) CAT activity in brain and plasma for Wistar rats.

After adding* Aronia* to water (*Aronia*-Yes) a decrease in the catalase activity in brain tissue was observed for all diet types (Figure 5). In case of the control diet (C−), the CAT-brain mean value decreased from 65.4 to 47.0 U/mg protein, in case of the fructose diet (F−) from 53.5 to 20.2 U/mg protein, and in case of the fat diet (Fa−) from 32.2 to 19.5 U/mg protein. It can be assumed that in this way the polyphenols in* Aronia* juice exercise a positive influence on the lipid peroxidation, and, therefore, the decreased CAT activity is a secondary symptom of the decreased fatty acid oxidation.

The catalase activity mean values for plasma, CAT-plasma, were much higher than for brain tissue, CAT-brain. Moreover, adding* Aronia* increased the catalase activity in all diet types. In case of the control diet (C−), the CAT-plasma mean value increased from 467 to 555 U/mg protein. A much bigger increase was observed in case of the fructose diet (F−) from 273 to 733 U/mg protein and in case of the fat diet (Fa−) from 248 to 687 U/mg protein. Increased CAT activity in plasma may suggest that an interaction of polyphenols present in* Aronia* juice with the CAT protein occurs. However, it should be further explained.

Decreased CAT activity and low concentration of sulfhydryl groups are probably associated with the transcriptional blockage of antioxidant enzymes. This involves reduction of the concentration of GSH, which is the main buffer of reduction in cells and protects proteins against the loss of biological functions. In our work, an increase in the SH group number and PON1 activity, as well as a decrease in CAT activity in brain tissue, was observed. This may be connected with a protective influence of* Aronia* on that tissue, especially when burdened with fat or fructose diet. In case of plasma, a decrease in PCG as well as the increase in CAT and PON1 activity for the fat diet was observed, which suggests that supplementation with* Aronia* may be beneficial for an organism burdened with the fat diet.

The current state of knowledge about the effects of supplementation with polyphenolic compounds in the state of physiological health, as well as in the case of carbohydrate loading, does not give the possibility to compare the results with other studies. Most of available literature concerning the in vivo studies presents results related to single-dose or chronic supplementation with polyphenols in toxic concentrations. This confirms the validity and relevance of the research.

Based on the conducted research, it was observed that feeding animals with carbohydrates or fats together with* Aronia* juice protects brain tissue against the effects of oxidative stress, caused by an increased number of free radicals. The conducted tests showed that supplementation with* Aronia* lowers the catalase activity, simultaneously with an increase in the PON activity in the brain. In case of the fructose and high fat diets with* Aronia* juice, the quantity of SH groups in brain tissue increased. In plasma, an increase in the CAT activity in case of the diets with* Aronia* juice was noted. In case of the high fat diet with* Aronia* juice, there was an increase in the PON activity in plasma. After administering* Aronia* juice, the number of SH groups in brain tissue increased, while in plasma a certain decrease in the SH groups was observed.* Aronia* juice did not significantly influence the number of carbonyl groups in brain tissue. In case of the high fat diet, a certain decrease in their number was noted. Big impact was observed in plasma, with the number of PCG significantly decreasing in each of the tested models.

## Figures and Tables

**Table 1:** The composition of experimental diets.

Components	Control diet (C) %	Fructose diet (F) %	Fatty diet (Fa) %
Starch	62	32	32
Casein	20	20	20
Oil	5.0	5.0	5.0
Lard	0	0	30
Fructose	0	30	0
Calcium carbonate	2.8	2.8	2.8
Ca_3_(PO_4_)_2_	2.9	2.9	2.9
Lecithin	1.0	1.0	1.0
NaCl	0.3	0.3	0.3
Cellulose	4.7	4.7	4.7
Minerals and vitamins mix.	1.0	1.0	1.0
MgO	0.07	0.07	0.07
K_2_SO_4_	0.23	0.23	0.23

**Table 2:** Activity of oxidative stress markers (PCG, FRAP, SH, PON1, CAT) marked in plasma and brain in Wistar rats.

Diet	*Aronia*	PCG nmol/mg protein	FRAP mM Fe^2+^/mg protein	SH nM/mg protein	PON1 U/mg protein	CAT U/mg protein
		Plasma				
C−	No	6.07 ± 1.23^A^	0.326 ± 0.065^A^	2.55 ± 0.26^AB^	363 ± 91^A^	467 ± 56^A^
F−	No	6.10 ± 1.30^A^	0.388 ± 0.075^A^	4.02 ± 1.23^A^	263 ± 73^AB^	273 ± 22^B^
Fa−	No	6.03 ± 0.69^A^	0.417 ± 0.146^A^	2.44 ± 0.64^AB^	187 ± 23^B^	248 ± 23^B^
C+	Yes	3.41 ± 1.90^B^	0.414 ± 0.047^A^	2.95 ± 1.12^AB^	240 ± 117^AB^	555 ± 62^A^
F+	Yes	1.48 ± 0.30^B^	0.428 ± 0.060^A^	3.26 ± 1.08^AB^	288 ± 74^AB^	733 ± 102^C^
Fa+	Yes	2.49 ± 0.86^B^	0.341 ± 0.043^A^	1.81 ± 0.85^B^	309 ± 73^AB^	687 ± 85^C^

		Brain				
C−	No	3.40 ± 1.47^A^	0.112 ± 0.015^A^	0.225 ± 0.091^AB^	31.8 ± 14.2^ABC^	65.4 ± 17.9^A^
F−	No	4.28 ± 1.37^A^	0.160 ± 0.044^AB^	0.317 ± 0.089^ABC^	20.7 ± 6.5^AB^	53.5 ± 20.6^AB^
Fa−	No	4.71 ± 1.51^A^	0.226 ± 0.017^B^	0.092 ± 0.013^A^	15.5 ± 6.0^A^	32.2 ± 13.7^BC^
C+	Yes	5.16 ± 2.22^A^	0.218 ± 0.048^B^	0.392 ± 0.107^BC^	37.1 ± 7.0^BC^	47.0 ± 16.6^AB^
F+	Yes	4.57 ± 0.91^A^	0.191 ± 0.032^B^	0.529 ± 0.229^BC^	41.9 ± 20.2^C^	20.2 ± 8.8^C^
Fa+	Yes	3.65 ± 1.00^A^	0.176 ± 0.044^AB^	0.206 ± 0.160^AB^	33.8 ± 8.9^ABC^	19.5 ± 5.5^C^

Data are presented as means from independent measurements ± standard deviation (SD). Different letters in the same columns indicate significant differences according to Tukey's test (*P* < 0.05).
